# Dynamics and virulence of *Enterobacteriaceae* reservoirs harboring *bla*_CTX-M_ group 1 in community wastewater

**DOI:** 10.1128/aem.00890-26

**Published:** 2026-09-08

**Authors:** Kieyarrah J. Dennis, Shuchen Feng, Melissa K. Schussman, Melinda J. Bootsma, Sandra L. McLellan

**Affiliations:** 1 School of Freshwater Sciences, University of Wisconsin-Milwaukee 14751, Milwaukee, Wisconsin, USA; University of Minnesota Twin Cities, St. Paul, Minnesota, USA

**Keywords:** *E. coli*, sewage, wastewater, antibiotic resistance, *bla*_CTX-M_, virulence genes

## Abstract

Extended-spectrum beta-lactamase (ESBL)-producing bacteria are ubiquitous and can cause serious infections. Here, we examined untreated community wastewater influent as a reservoir for *bla*_CTX-M_ group 1 organisms and their virulence potential. Raw influent samples (*n* = 268) were collected from four wastewater treatment plants (WWTPs) representing dense urban populations. We observed that *bla*_CTX-M_ group 1 levels were high at all WWTPs and only ~1–2 log_10_ lower and not correlated to common human-specific microbiome fecal markers, Lachno3 and HF183, indicating a lack of connection to human fecal inputs. Concentrations of *bla*_CTX-M_ group 1 genes and markers for presumptive host organisms *Escherichia coli* and *Klebsiella pneumoniae* were influenced by travel time and season. Amplicon sequencing revealed high diversity of *bla*_CTX-M_ group 1–9 genes, with 63% belonging to group 1. Selective culture and 16S rRNA gene sequencing showed *bla*_CTX-M_ group 1 isolates were 26% *E. coli*, 26% *K. pneumoniae*, 40% other *Enterobacteriaceae*, and 8% *Aeromonas*. Overall, *E. coli* averaged 3.6E7 cells/L, with 3% of all *E. coli* found to contain *bla*_CTX-M_ group 1. Whole-genome sequencing of *bla*_CTX-M_ group 1 *E. coli* from wastewater revealed resistance and virulence gene profiles similar to clinical isolates and distinct from other wastewater ESBL-resistant and non-resistant *E. coli*. Interpretation of wastewater data needs to consider both the existence of environmental reservoirs that contain potentially pathogenic organisms and the strong influence the dynamics of the conveyance system can have on final concentrations measured at the WWTP.

**IMPORTANCE:**

The CTX-M enzyme family is highly abundant in nosocomial, community, and environmental settings and is leading to treatment of infections with carbapenem antibiotics, a last-line therapeutic option. The progressive increase of the clinically relevant *bla*_CTX-M_ group 1 resistance genes in the human population warrants investigation, particularly to understand the establishment and dynamics of environmental reservoirs. This study utilized molecular and culture methods to gain insight into the possible origin, abundance, and dynamics of *bla*_CTX-M_ group 1 genes in untreated wastewater influent samples. We found extremely high levels of these genes, with *Escherichia coli* as a major host organism that closely resembled clinical strains, suggesting they are seeded and propagate in sewer pipe systems. The significance of our research is in developing approaches to monitor antimicrobial resistance reservoirs in community wastewater, which could shed light on global burdens and potential transmission cycles and indicate increasing inputs of clinically relevant strains originating from human populations.

## INTRODUCTION

Bacterial resistance to antibiotic therapies is a serious global public health concern that continues to increase in severity, frequency, and complexity (1, 2). Extended-spectrum β-lactamases (ESBLs) are the most abundant resistance enzymes found, and beta-lactam antibiotics are the most widely used (3, 4). Restructuring antibiotic distribution for medicinal therapies will not significantly affect the state of the issue without considering factors such as environmental spread, environmental reservoirs for organisms that carry antibiotic resistance genes (ARGs), and the viability of ARGs found outside of hospitals (5, 6). One of the most present and clinically important ESBL subgroups is cephalosporinases, and their most impactful enzyme family is CTX-M (7). One of the most clinically relevant and abundant CTX-M enzymes is CTX-M-15, which is in the group 1 subfamily (8).

Untreated wastewater influent offers a means to assess the human population for viruses and pathogenic bacteria, as well as antibiotic resistance genes of clinical importance (9). It can also be an effective method for evaluating potential environmental reservoirs of antibiotic resistance genes (6). Studies using wastewater samples have been successful for monitoring pathogens like SARS-CoV-2 (10, 11). However, making concrete conclusions about human carriage of antibiotic-resistant bacteria from wastewater samples is difficult because non-target free-living organisms can contribute a substantial background signal (12). When sampling wastewater and urban wetland environments, researchers have observed *bla*_CTX-M_ variants not cataloged in GenBank, indicating that these enzymes may represent a mobile reservoir of potentially medically important resistance genes (13). CTX-M genes evolved in multiple species belonging to the environmental bacterial genus *Kluyvera* and spread to other species through plasmids, eventually reaching a pathogenic host (14, 15). This enzyme family and their genes have been closely associated with pathogenic bacteria in the *Enterobacteriaceae* family, namely, *Escherichia coli* and *Klebsiella pneumoniae* in the western world since the early 2000s (16), and are the most common in exhibiting ESBL resistance (17), which is concerning as they are also common commensal organisms with flexible genomes (18–20). As a pathogen, *K. pneumoniae* is opportunistic and infects/colonizes the immunocompromised, hospital inpatients, and those using multiple antibiotic therapies (18). *E. coli* is one of the most common commensal and pathogenic bacteria in humans and agricultural animals and is associated with both nosocomial and community-onset infections (19).

We analyzed untreated wastewater influent samples from four wastewater treatment plants (WWTPs), including influent from two WWTPs with different characteristics and within the same city, to examine how conveyance system dynamics might affect antibiotic resistance monitoring. We specifically investigated if the *bla*_CTX-M_ group 1 gene signal could be accounted for by human inputs versus environmental reservoirs that may have been formed from seeding of clinical isolates or represent organisms not associated with human fecal inputs. We used whole-genome sequencing of wastewater isolates and large comparisons of clinical isolates to explore the origin and virulence of the CTX-M carrying organisms in wastewater.

## RESULTS

We quantified *bla*_CTX-M_ group 1 levels using droplet digital PCR (ddPCR) in untreated wastewater influent samples obtained from two WWTPs: Jones Island (JI) and South Shore (SS) in Milwaukee, WI, USA, over a 10-year period (2013–2023) and used parameters from the conveyance systems and WWTPs for in-depth analysis. In addition, we quantified samples from Green Bay, Wisconsin (PLT), and Racine, Wisconsin (RAC), obtained in 2023. We also measured concentrations of markers targeting two human fecal-specific bacteria (HF183 and Lachno3) and two presumptive host organisms for *bla*_CTX-M_ group 1, *E. coli* (*uid*A marker) and *K. pneumoniae* (*khe* marker) using quantitative PCR (qPCR). The plants collect untreated wastewater from numerous hospitals and provide sanitation services for 110,000–500,000 people each but with different travel times (Table 1).

**TABLE 1:** Characteristics of wastewater catchments, infrastructure, and influent samples^*b*^

WWTP	Population	Flow (MGD)	BOD (mg/L)	Mean travel time (hours)	System type	No. of samples
Milwaukee, Jones Island	470,007	17–380	74–480	7.5	Partially combined	116 + 25 daily
		97	265			
Milwaukee, South Shore	615,934	30–305	80–930	22	Separated	87
		92	318			
Racine	110,000	11–61	NA^*a*^	NA^*a*^	Separated	20
		20				
Green Bay PLT	189,000	14–72	NA^*a*^	NA^*a*^	Separated	20
		26				

^*a*^ NA, not available.

^*b*^ Samples were collected from 2013 to 2023. Flow and biological oxygen demand (BOD) are reported as a range of minimum to maximum (top numbers) and daily average (bottom number).

### *bla*_CTX-M_ group 1 and organism abundance in untreated wastewater influent

**Fig 1:**
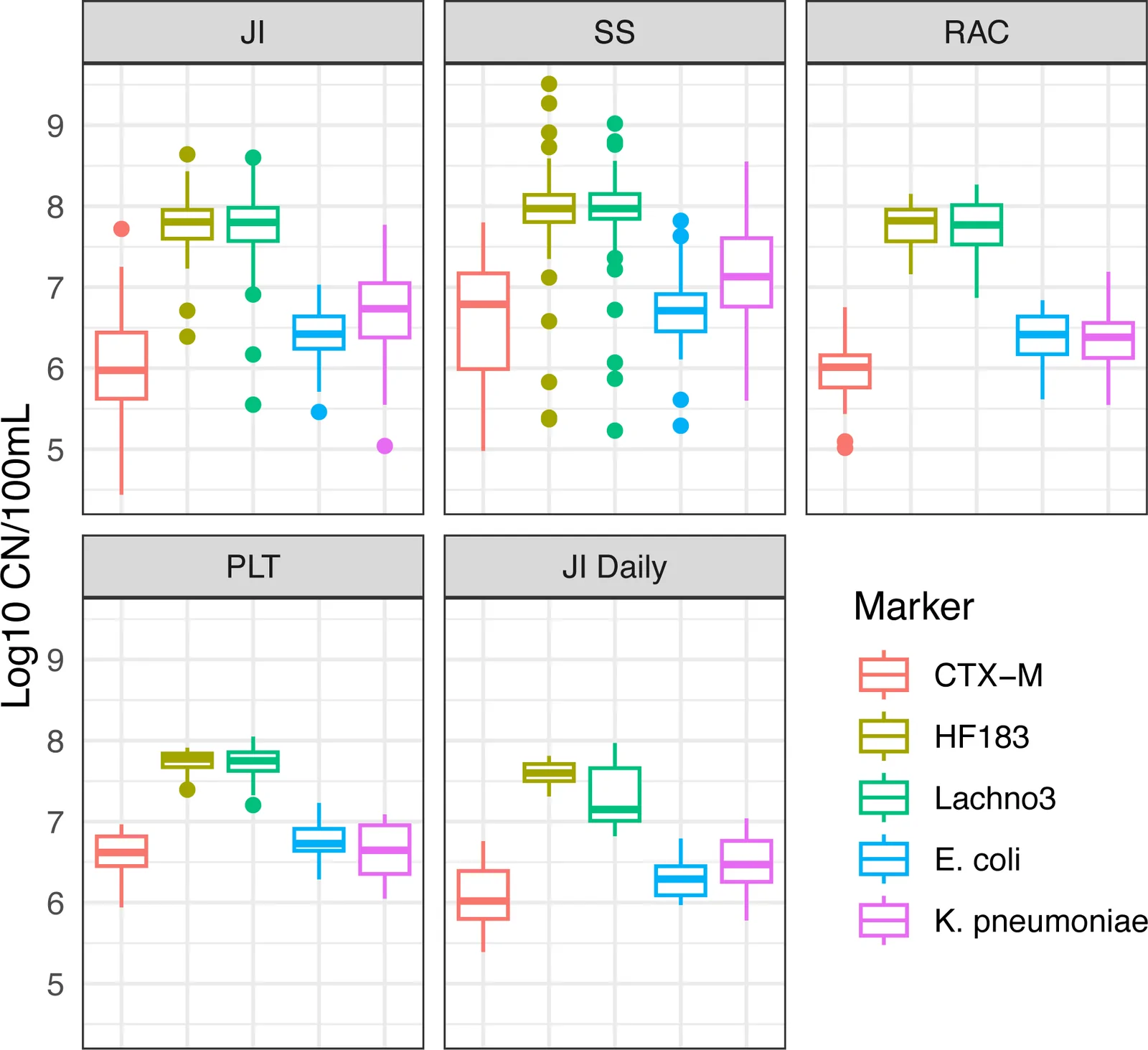
Log_10_ values of *bla*_CTX-M_ group 1, *E. coli*, *K. pneumoniae,* and human-specific markers HF183, Lachno3 in raw influent from the Jones Island (JI) WWTP (*n* = 116), South Shore (SS) WWTP (*n* = 87) from 2013 to 2023, Racine (RAC) WWTP (*n* = 20) from 2023, Green Bay (PLT) (*n* = 20) from 2023, and daily JI samples (*n* = 25) from July 2024. Boxplot line represents the median, whiskers represent the 25th (lower) and 75th (upper) interquartile ranges, and standalone dots represent outliers.

The *bla*_CTX-M_ group 1 gene concentrations were found to be on average 5.9 ± 0.6 (mean ± standard deviation) Log_10_ CN/100mL at JI and 6.5 ± 0.7 Log_10_ CN/100mL at SS. These numbers are considered high due to their proximity to the human marker values (HF183 and Lachno3), which were coupled to each other and were only on average ~1.5 to 2 orders of magnitude higher than the *bla*_CTX-M_ group 1 values (Fig. 1; Table S1). Human fecal markers (HF183 and Lachno3) showed highly similar levels in raw influent from JI and SS (Fig. 1), and when considering parameters such as flow and population, the marker levels per person per day were nearly identical across WWTPs. On average, *E. coli* levels were only ~2-fold higher than *bla*_CTX-M_ group, and *K. pneumoniae* ~6-fold higher than *bla*_CTX-M_ group 1. Both Racine and Green Bay, sampled across a shorter time period, showed similar patterns, demonstrating that high *bla*_CTX-M_ group 1 levels are widespread in wastewater in urban areas. We further examined if levels of *bla*_CTX-M_ group 1 and *E. coli* and *K. pneumoniae*, as presumptive host organisms, were accumulating over time. The *bla*_CTX-M_ group 1 had a weak but significant upward trend in JI and SS raw influent, whereas the presumptive host organisms *E. coli* and *K. pneumoniae* showed no trend of increasing levels over the 10-year period (Fig. S1).

### Correlations of *bla*_CTX-M_ groups 1–9 with bacterial markers and WWTP-associated wastewater parameters

**Fig 2:**
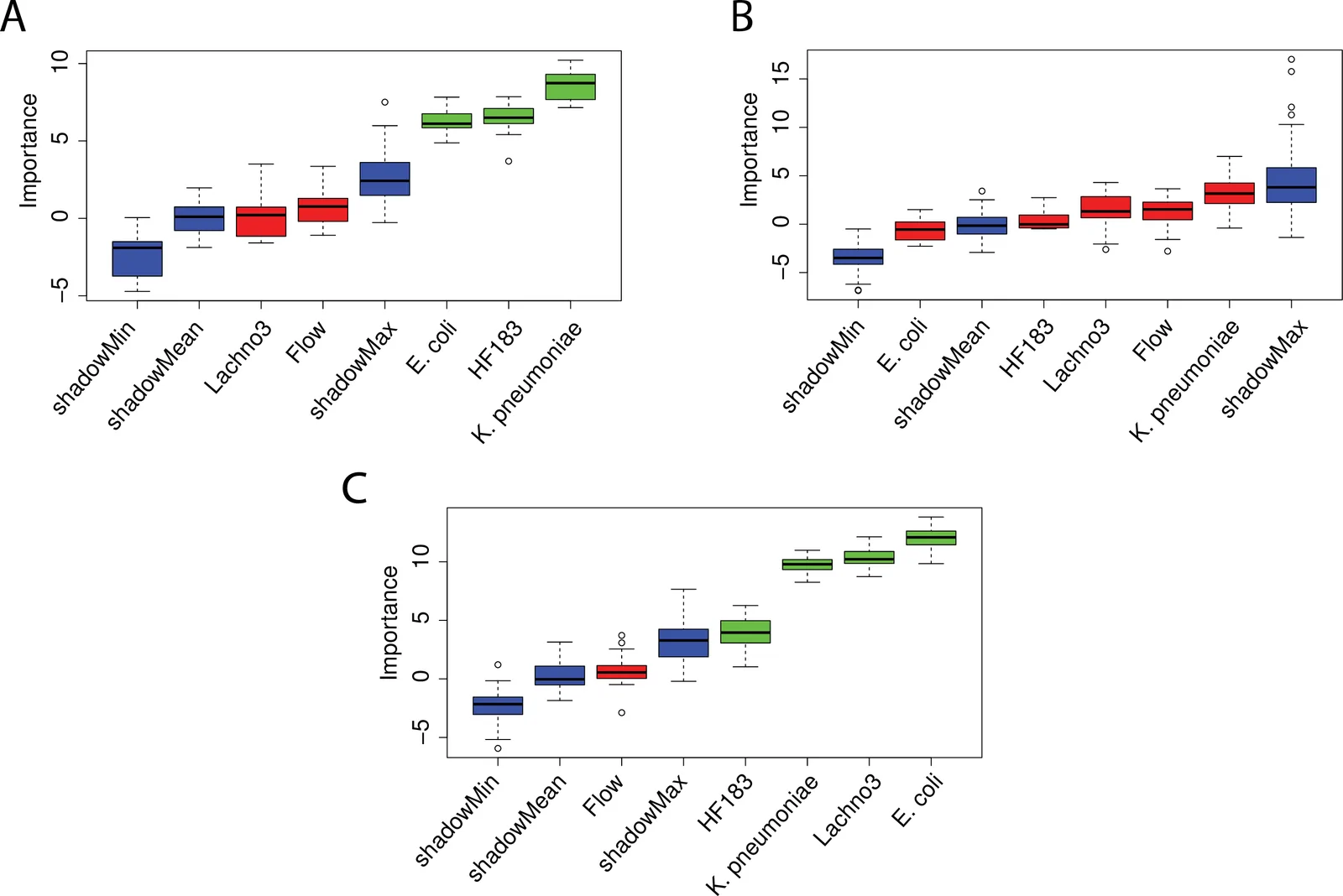
CTX-M correlation with organism markers and WWTP-associated wastewater parameters. Boruta analysis identified the relevance of factors on *bla*_CTX-M_ group 1–9 concentrations in wastewater from (**A**) JI (*n* = 116), (**B**) SS (*n* = 87), and (**C**) JI daily samples (*n* = 25). The blue boxes represent the shadow features, which are randomized versions of the original features used as a reference to establish the importance threshold and show values of “shadowMin,” “shadowMean,” and “shadowMax,” which represent a shadow feature’s minimal, average, and maximum *Z*-score decided by Boruta, respectively. Features with importance metrics exceeding the shadowMax value are classified as important (green boxes), whereas those below the shadowMax value are classified as not important (red boxes).

We used Boruta analysis to assess the relationship of all relevant variables in our data set to our specific central variable (*bla*_CTX-M_ group 1) (Fig. 2). In the influent sewage samples collected from 2013 to 2023 at the JI WWTP, *E. coli*, HF183, and *K. pneumoniae* were determined to be important to *bla*_CTX-M_ group 1 concentrations, while flow and Lachno3 were not. However, in the daily JI samples, flow was the only factor found to not significantly influence *bla*_CTX-M_ group 1 levels. In contrast, for SS WWTP, which has longer travel times, none of the analyzed variables were observed to be important to *bla*_CTX-M_ group 1 levels.

We examined the hypothesis that the *bla*_CTX-M_ group 1 genes originated from human inputs by examining how closely the *bla*_CTX-M_ group 1 and human markers were correlated. The two human markers are host-associated fecal anaerobes, and they serve as a good representation for current human fecal inputs as they are expected to have limited survival outside of their host. We found that the human markers HF183 and Lachno3 mirrored one another in concentration patterns and had a fairly strong positive relationship in JI (rho = 0.68, *P* < 0.05) and SS (rho = 0.84, *P* < 0.05) but low correlation to *bla*_CTX-M_ group 1 (Fig. S1). The presumptive host bacterial markers, *E. coli* and *K. pneumoniae*, also had a fairly strong positive correlation to each other (rho = 0.75, *P* < 0.05), but both organisms had a lower correlation to the human markers. The *bla*_CTX-M_ group 1 levels had a low correlation to *E. coli* and *K. pneumoniae* in JI (rho = 0.34 and 0.33, *P* < 0.05, respectively) and no significant correlation in SS. JI daily correlations among host markers and *bla*_CTX-M_ group 1 were notably stronger, suggesting that these relationships are more consistent in a short time frame when conditions in the conveyance system were more stable.

### Temperature effects on *bla*_CTX-M_ group 1 and bacterial markers

**Fig 3:**
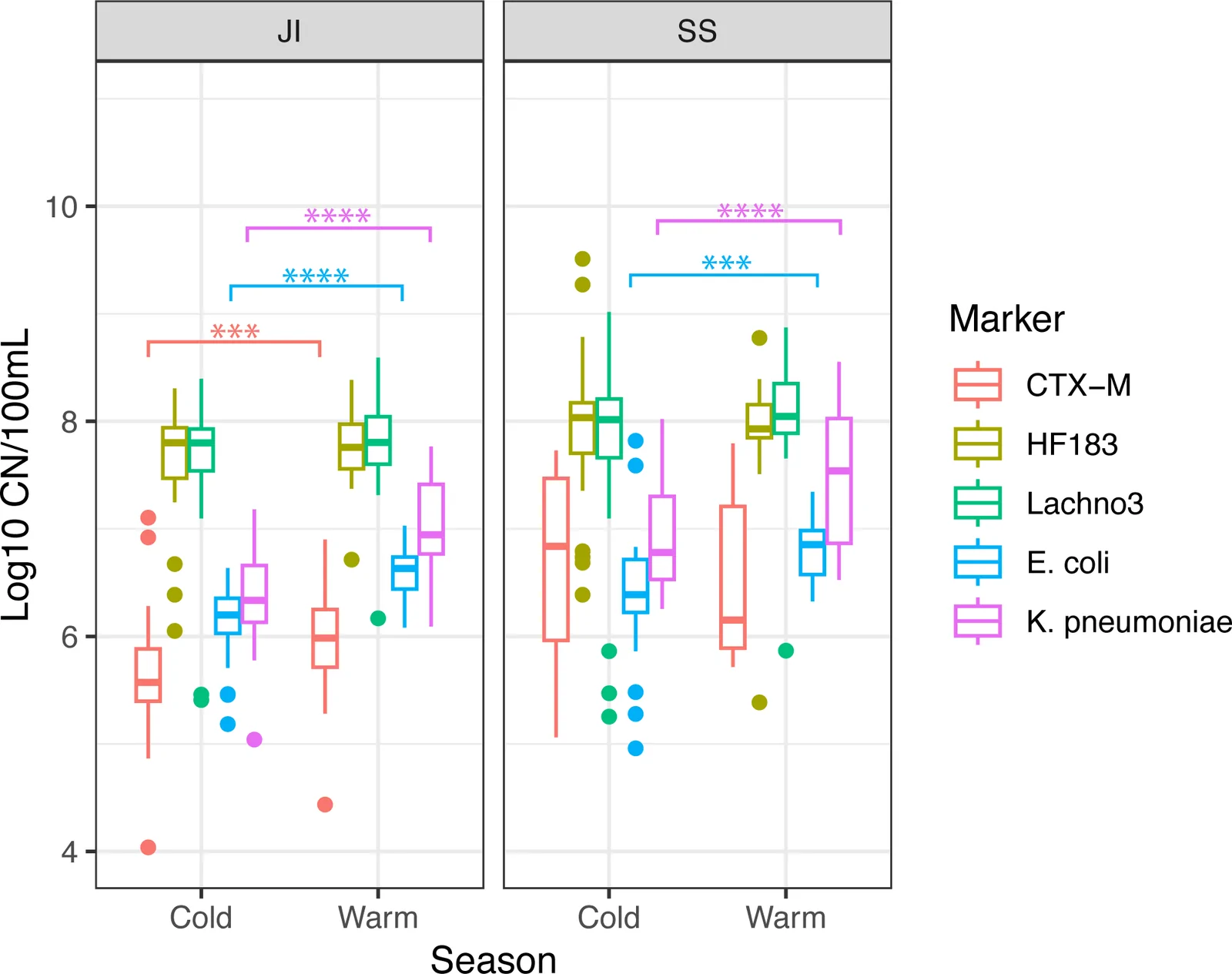
Boxplot showing various marker abundances comparing cold (February–May) and warm (August–November) collection times at JI (*n* = 66) and SS (*n* = 55) over the study period. Boxplot line represents the median; whiskers represent the 25th (lower) and 75th (upper) interquartile ranges; standalone dots represent outliers. ***P < 0.001, ****P < 0.0001.

To further explore how temperature impacted marker concentrations, we conducted a seasonal analysis. Temperature analysis was done by binning samples into cold (February, March, April, and May) and warm (August, September, October, and November) categories based on actual temperatures present in the conveyance systems (21, 22). The human marker concentrations were not significantly different (increased or decreased) in cold versus warm months at either WWTP (Fig. 3; Table S2). The *bla*_CTX-M_ group 1 abundances increased nearly twofold from cold to warm seasons at JI WWTPs, whereas there was no significant difference at SS WWTP. The presumptive host bacterial markers had the greatest increase in concentrations from cold to warm sampling periods at both WWTPs with a twofold increase of *E. coli* and a three- to fourfold increase of *K. pneumoniae*.

### Sequencing of *bla*_CTX-M_ amplicons

**Fig 4:**
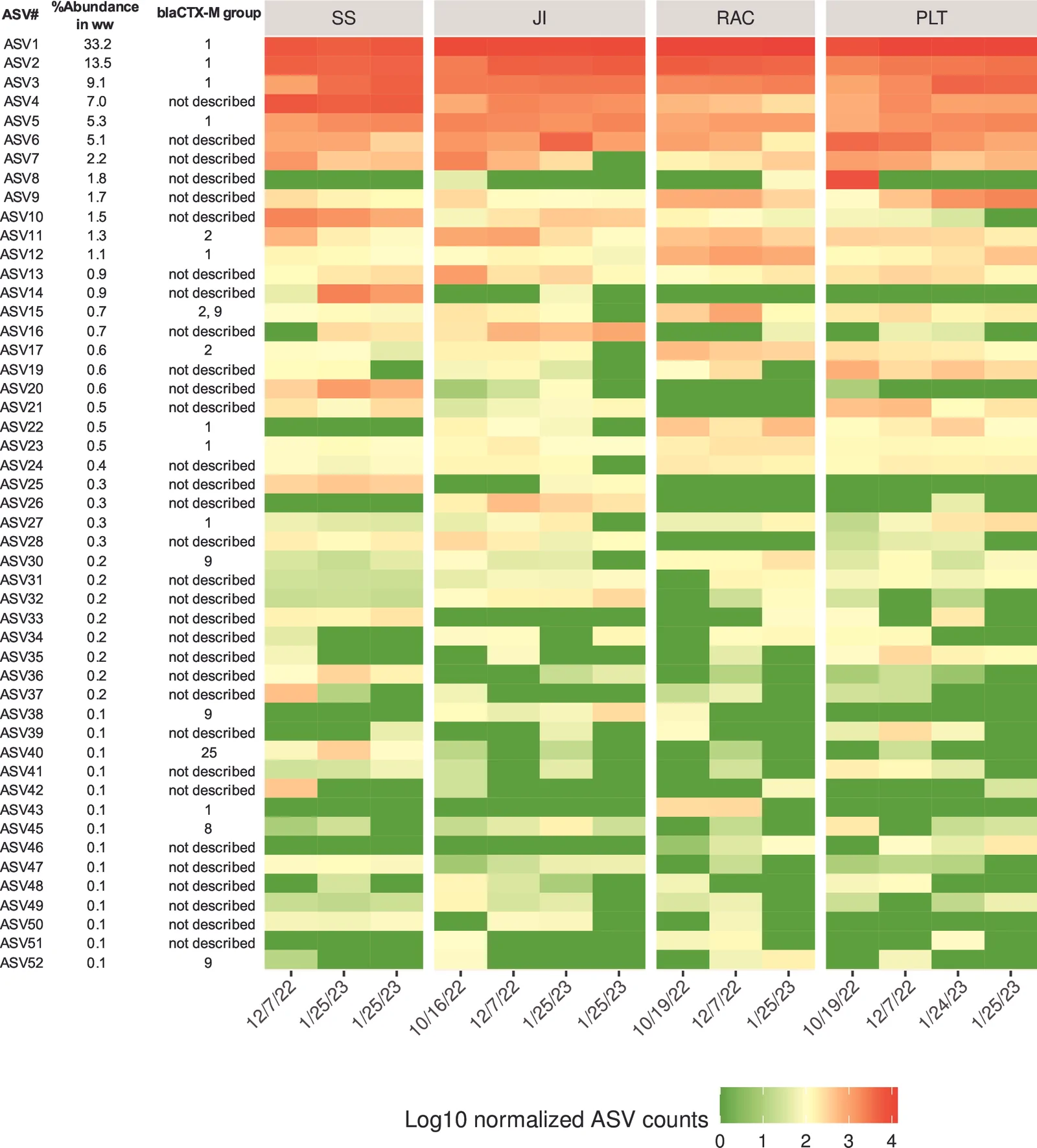
Distribution of sequenced *bla*_CTX-M_ group 1–9 ASVs from raw influent from four WWTPs; South Shore (SS), Jones Island (JI), Racine (RAC), and Green Bay (PLT). Samples are normalized to the average reads recovered in a sample, which was 30,000. ASVs 18, 29, and 44 are non-specific amplification of the *bla*_CTX-M_ group 1–9 assay and are excluded. ASV40 is classified to match *bla*_CTX-M_ group 25 and is a close match to the *bla*_CTX-M_ group 1–9 forward and reverse primers used to generate the amplicons. ASVs not matching CARD database *bla*_CTX-M_ genes 100% are listed as not described.

We performed amplicon sequencing for the *bla*_CTX-M_ group 1–9 PCR products in 14 raw influent samples over a range of seasons. We included samples from Green Bay and Racine, Wisconsin, in addition to JI and SS, to assess the diversity of *bla*_CTX-M_ genes across a larger number of WWTPs. Each sample was normalized to a total of 30,000 reads (mean reads number for all samples), and amplicon sequence variant (ASV) profiles were assessed (Fig. 4). The top 3 ASVs all belonged to group 1 and accounted for 56% of all reads, with several other ASVs matching group 1 also present (an additional 7.4% of all reads). ASVs matching other *bla*_CTX-M_ groups only comprised 3.2% of the reads, with the remaining having no exact match to *bla*_CTX-M_ group genes but were closely related, suggesting that they are previously undescribed *bla*_CTX-M_ genes. The distribution of common *bla*_CTX-M_ group 1–9 ASVs was fairly uniform across plants and across seasons. Only three of the minor ASV sequences (<1% abundance; ASVs 18, 29, and 44) were non-specific for *bla*_CTX-M_ and were not considered in further analysis.

We then compared ASVs occurring >1% with the National Center for Biotechnology Information (NCBI) nucleotide database using BLAST to assess the organism where these genes have been previously reported. We found that these amplicon types are related to species associated with both clinical and environmental sources (Table 2; Table S3).

**TABLE 2:** Exact NCBI Nucleotide database matches of the top *bla*_CTX-M_ group 1–9 amplicon sequences^*a*^^,^^*c*^

ASV^*b*^	% Abundance in WW	Organisms (% of exact matches)											
		*A.* *baumannii*	*A.* *caviae*	*C.* *freundii*	*E.* *cloacae*	*E.* *coli*	*K.* *pneumoniae*	*R.* *ornithinolytica*	*Kluyvera* spp.	*P.* *aeruginosa*	*S.* *enterica*	Uncultured	Other
**ASV1**	**33.2**	0.45	0.11	0.71	1.1	35	49	0.27	0.20	0.42	1.3	1.4	10
**ASV2**	**13.5**					41	27			9.1		18	4.6
**ASV3**	**9.1**			6.3		13				6.3		75	0
ASV4	7.0		5.6			17	5.6					56	17
**ASV5**	**5.3**					12						76	12
ASV6	5.1											100	0
ASV7	2.2											100	0
ASV11	1.3	5.0			5.0	5.0			30		55		0
**ASV12**	**1.1**				33						17	17	33
ASV30	0.24		0.09	0.51	0.28	62	23	0.42	0.05		5.5	0.19	8.6
ASV38	0.14			3.1	2.6	32	33	0.44	0.87		3.9	0.44	24
ASV52	0.10				31	7.7	15		15				31

^*a*^ The top 12 most abundant ASVs (>1%) and select minor ASVs are shown.

^*b*^ Exact matches are defined as 100% identity across the entire query of the CTX-M amplicon. ASVs matching *bla*_CTX-M_ group 1 are shown in bold. ASV8, ASV9, and ASV10 were present at 1%–2% relative abundance but did not have a match in NBCI. These top 12 ASVs accounted for 83% of the reads. Select minor ASVs are shown as they were recovered in cultured isolates.

^*c*^ *A. baumannii, Acinetobacter baumannii; A. caviae, Aeromonas caviae; C. freundii, Citrobacter freundii; E. cloacae, Enterobacter cloacae; R. ornithinolytica, Raoutella ornithinolytica; P. aeruginosa, Pseudomonas aeruginosa*.

### Culture of CTX-M positive isolates from community wastewater

The poor correlation of *bla*_CTX-M_ group 1 gene levels with human markers (HF183 and Lachno3) suggested *bla*_CTX-M_ signals could be associated with both human and environmentally derived organisms. While correlations were stronger with presumptive hosts *E. coli and K. pneumoniae* (which can be from human and environmental sources), we examined if other organisms not directly derived from human fecal inputs could account for the high signal. We also aimed to compare the organisms frequently identified in GenBank (Table 2) with what organisms containing *bla*_CTX-M_ group 1 were actually recovered in wastewater.

A total of 48 colonies were isolated on ESBL CHROMagar from raw influent samples collected in July 2025 from JI and SS (Table S4). A total of 22 isolates were found to have *bla*_CTX-M_ groups 1–9, suggesting other colonies were resistant to the antibiotic selection by alternate resistance mechanisms. The majority of the *bla*_CTX-M_ isolates (70%) were identified as group 1 (*n* = 15) and matched ASV1 (Table 3) and included *E. coli* (*n* = 4), *K. pneumoniae* (*n* = 4), other *Enterobacteriaceae* (*n* = 6), and *Aeromonas* (*n* = 1) identified as the host organisms. The remaining 30% of the *bla*_CTX-M_ isolates were group 9 and were primarily *E. coli* (*n* = 6), with one isolate most closely related to *Klebsiella*, *Raoultella*, or *Kluyvera* (*n* = 1). The recovery of primarily *E. coli* and *K. pneumoniae* was consistent with the most distribution of previously reported organisms in GenBank.

**TABLE 3:** Comparison of ASVs amplified directly from wastewater samples and cultured isolates

ASV	Relative abundance of ASV (%)	CTX-M enzyme group	No. of isolates screened	Isolate identification (no. of organisms)
ASV_1	33.2	1	*n* = 15	*Escherichia* (4), *Klebsiella* (4), *Klebsiella/Raoultella* (4), *Enterobacter* (2), *Aeromonas* (1)
ASV_30	0.2	9^*a*^	*n* = 5	*Escherichia* (5)
ASV_38	0.1	9	*n* = 1	*Escherichia* (1)
ASV_52	0.1	9	*n* = 1	*Klebsiella*/*Raoultella*/*Kluyvera* (1)

^*a*^ Rare matches in CARD database to group 1, 98% match to group 9, qPCR for group 1 negative.

We also evaluated whether the abundance of *bla*_CTX-M_ group 1 positive isolates recovered accounted for the signal detected in wastewater to determine if the ESBL CHROMagar was recovering the majority of *bla*_CTX-M_ group 1 positive organisms. We estimated we recovered 5.68 Log_10_ CFU per 100 mL for JI and 5.43 Log_10_ for SS of *bla*_CTX-M_ group 1 isolates on the selective agar, which was within an order of magnitude of our ddPCR signal and concluded we were not missing a major group of organisms responsible for the high signals.

### Beta-lactamase ARG and virulence gene profiles of *bla*_CTX-M_ wastewater *E. coli* isolates

We performed whole-genome sequencing on 24 *E. coli* selected for cefotaxime resistance. We examined the resistance and virulence gene profiles of these isolates and compared them with *E. coli* isolated with non-selective media and clinical isolates from sources including infections. Similar to our results on CHROMagar, 9 of 24 isolates from ESBL selective media were *bla*_CTX-M_ group 1 positive, with the other isolates having a resistance mechanism different than *bla*_CTX-M_. The wastewater *bla*_CTX-M_ group 1 isolates were nearly evenly divided as CTX-M-15 and CTX-M-55, which was disproportional to what was observed from our clinical isolate collection (Table S5). The overall resistance profiles of beta-lactamase genes demonstrated a progression of accumulation, with the highest abundance observed inclinical genomes, followed in descending order by wastewater ESBL *bla*_CTX-M_+, wastewater ESBL *bla*_CTX-M_−, and wastewater non-selective isolates (Table 4), generally with multiple copies of the beta-lactamase genes found. Interestingly, the *E. coli* recovered with non-selective media all contained ampC, and 22 of the 24 isolates had 2 copies of this gene.

**TABLE 4:** Beta-lactamase ARG profiles and common STs of *E. coli* in clinical and wastewater isolates

ARG^*a*^	No. of genomes positive per 20 genomes assessed (total copies found)^*b*^			
	Clinical (ST167, ST410, ST361, ST131, ST69, ST38, ST95)	Wastewater ESBL *bla*_CTX-M_+ (ST167, ST155, ST361)	Wastewater ESBL *bla*_CTX-M_– (ST69, ST38, ST155)	Wastewater non-selective (ST58, ST95, ST141, ST69)
ampC	6 (41)	20 (22)	15 (20)	20 (38)
*bla*_CMY-2_	4 (9)	7 (11)	4 (4)	0
*bla*_CTX-M_	14 (28)	20 (20)	0	0
*bla*_KPC_	1 (2)	0	0	0
*bla*_NDM_	16 (31)	4 (4)	0	0
*bla*_OXA-1_	8 (15)	4 (7)	0	0
*bla*_OXA-10_	0.3 (0.6)	0	0	0
*bla*_OXA-48_	2 (3)	2 (2)	0	0
*bla*_TEM_	9 (19)	13 (16)	3 (3)	0
*bla*_VIM_	2 (3)	0	0	0
penP	2 (3)	0	0	0
Total	64 (155)	70 (82)	22 (27)	20 (38)

^*a*^ ARG are normalized to counts per 20 genomes in each group for comparison across groups. Original number of *E. coli* genomes analyzed included clinical genomes (*n* = 72), wastewater ESBL selected *bla*_CTX-M_+ (*n* = 9), wastewater ESBL selected *bla*_CTX-M_− (*n* = 15), and wastewater non-selective (*n* = 24).

^*b*^ Values are normalized to 20 genomes and reported as ARG-positive genomes (total ARG copies). STs listed are ordered by abundance in each group. Underlined STs are shared between clinical and wastewater sources. See Data Set S1 for detailed information.

We identified a total of 64 sequence types (STs) from all clinical and wastewater *E. coli* genomes (Table 4, Data Set S1). The clinical genomes we used in this analysis (*n* = 72) had the most abundant commonly reported clinical STs such as ST167 (*n* = 15), ST410 (*n* = 10), ST361 (*n* = 8), and ST131 (*n* = 4). Wastewater *E. coli* shared a limited number of STs with the clinical genomes. We identified two ST167 and one ST361 from the nine wastewater ESBL *bla*_CTX-M_+ selective isolates, and three other STs (ST38, ST69, and ST101) in the collection of 15 wastewater ESBL *bla*_CTX-M_– isolates. Non-selective wastewater genomes (*n* = 24) only had two STs that were present in clinical sources (ST69 and ST95).

**Fig 5:**
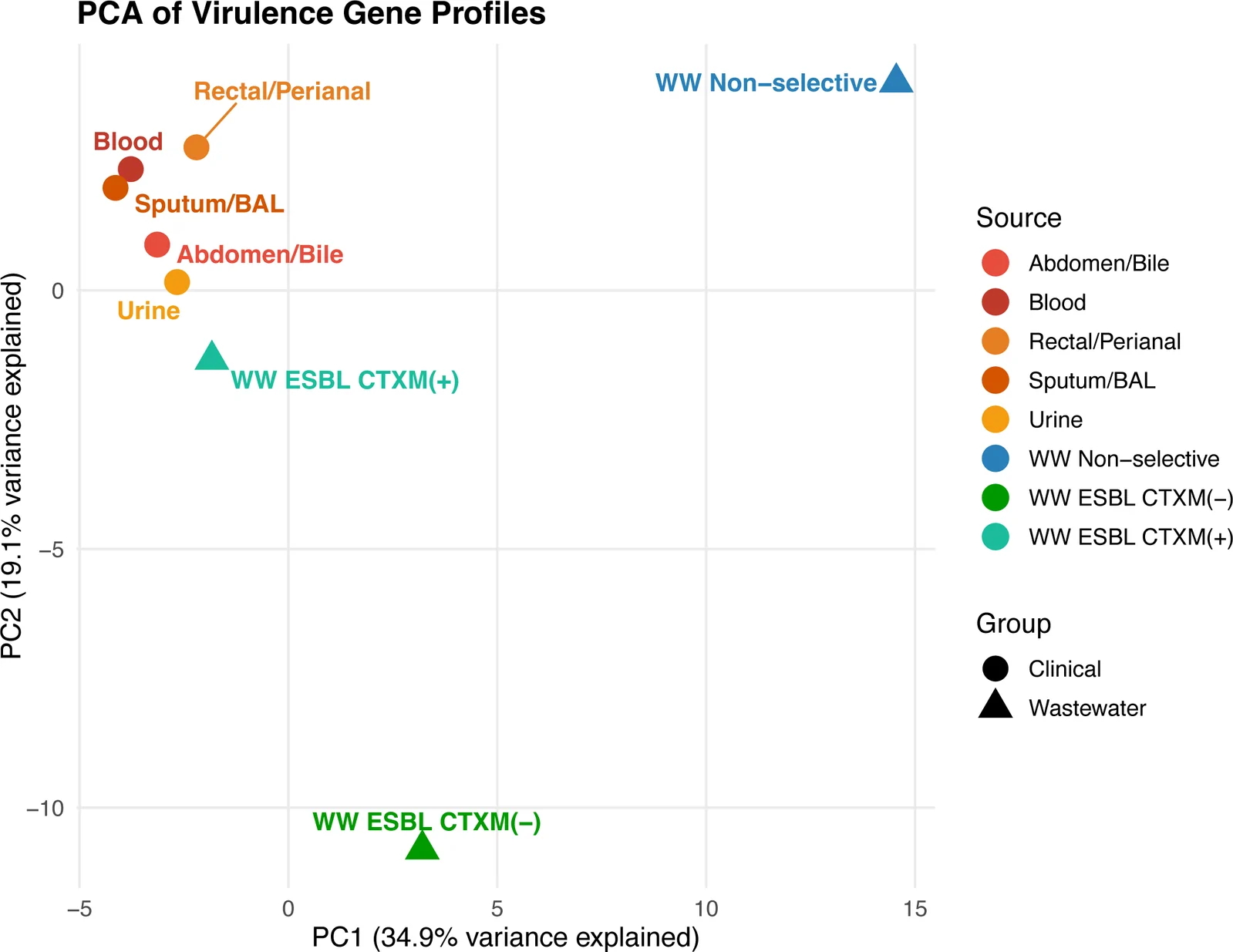
PCA plot of virulence gene occurrence in *bla*_CTX-M_+ clinical samples (abdomen/bile, *n* = 8; blood, *n* = 5; rectal/perianal, *n* = 7; sputum/BAL, *n* = 2; urine, *n* = 19), ESBL *bla*_CTX-M_+ group 1 (*n* = 9) and ESBL *bla*_CTX-M_– (*n* = 15) wastewater selected isolates, and wastewater non-selective isolates (*n* = 24).

**Fig 6:**
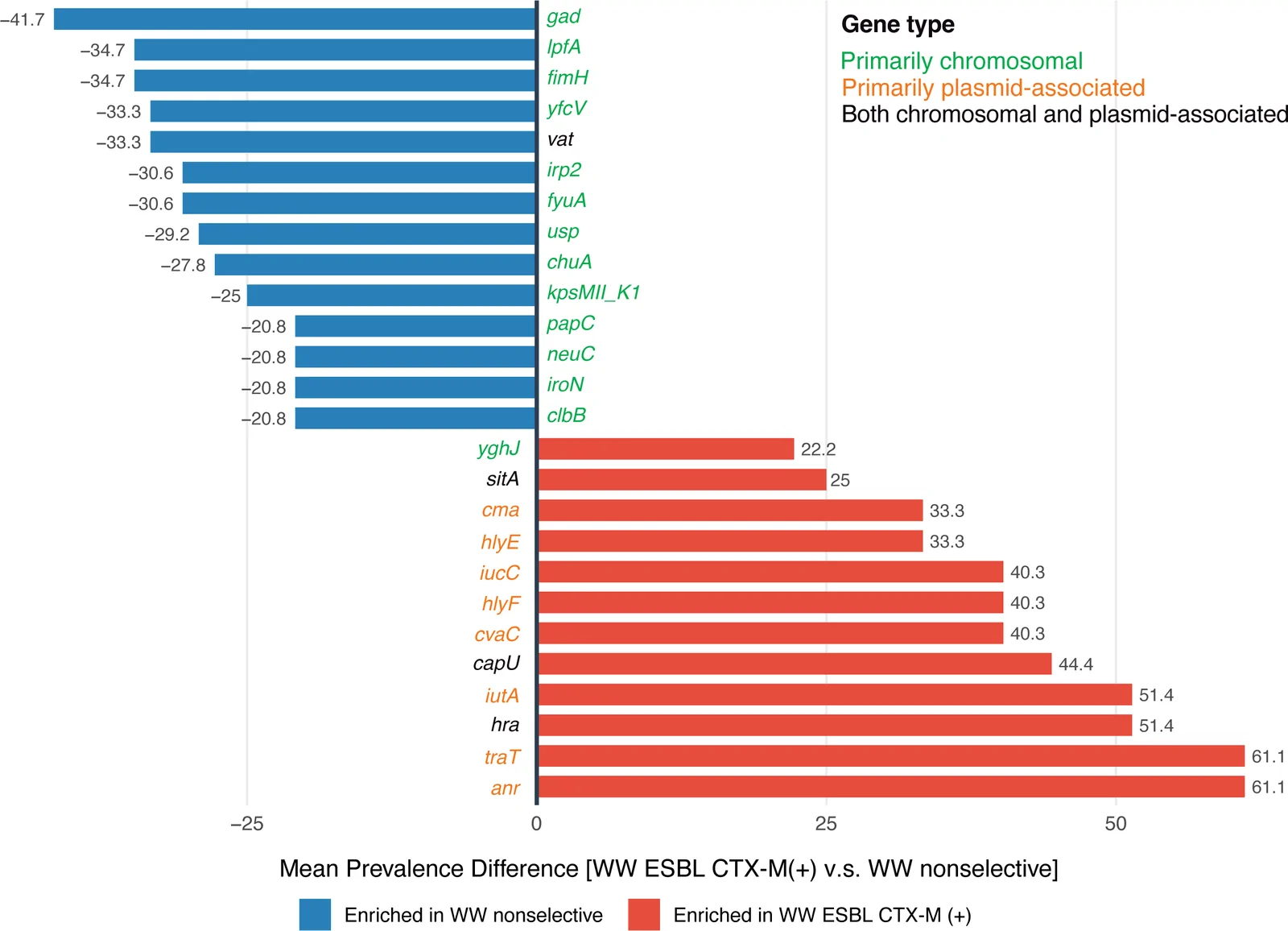
Top 12 differentially enriched virulence genes in wastewater ESBL *bla*_CTXM_ group 1 + and wastewater non-selective *E. coli* genomes.

The virulence gene profiles show a clear separation of clinical and ESBL *bla*_CTX-M_+ wastewater isolates from the ESBL *bla*_CTX-M_– isolates and the non-selective wastewater isolates (Fig. 5). All of the ESBL *bla*_CTX-M_+ isolates were group 1. *E. coli* from all sources shared similar overall virulence gene abundances but with different gene collections. Within the wastewater group, the progression of increasing ARG content (Table 4) mirrored the separation of the three groups of *E. coli* based on virulence gene content, with a different enrichment of virulence genes for ESBL *bla*_CTX-M_+ genomes, ESBL *bla*_CTX-M_– genomes, and non-selective genomes (Fig. S3). We found that virulence genes enriched in non-selective genomes are primarily chromosomal (e.g., *chuA*, *gad*, and *IpfA*), while those enriched in ESBL selected genomes or clinical isolates are mostly plasmid-associated (e.g., *traT*, *anr*, *hra*, and *iutA*) (Fig. 6). Taken together, these data indicate that the virulence profiles of clinical isolates and *bla*_CTX-M_ group 1 wastewater *E. coli* are mainly associated with antibiotic resistance on mobile genetic elements such as plasmids and distinctive populations of progressively resistant strains are introduced into the wastewater system, highlighting how community wastewater are a potential environmental reservoir where strains with high virulence and resistance traits can persist and circulate.

## DISCUSSION

Our results support the hypothesis that clinically relevant organisms carrying *bla*_CTX-M_ group 1 are seeded into the wastewater system and propagate, creating reservoirs that are disproportionally high compared to human contributions. Even considering colonization rates of healthy individuals that could be 0.1% to as high as 6% (23–25), extremely high levels of *bla*_CTX-M_ group 1 are inconsistent with the amount of estimated human fecal input. Coupled with the evidence that conditions in the conveyance system can cause large fluctuations in concentrations without concurrent fluctuations of human fecal markers, these data demonstrate that environmental reservoirs contribute to *bla*_CTX-M_ group 1 signals detected in wastewater at the plant.

Further, our long-term wastewater influent data set suggests increasing reservoirs of *bla*_CTX-M_ group 1 over time, which may be a result of more colonization of individuals and therefore more seeding into the system. Importantly, our genomic sequencing of *E. coli* demonstrated clear stepwise groups of progressively resistant and virulent organisms that parallel acquisition of *bla*_CTX-M_ group 1, which is consistent with selection in the clinical setting with subsequent seeding and propagation, rather than plasmid transfer among resident organisms in the system itself.

### High *bla*_CTX-M_ group 1 abundance and relationship to human fecal contributions

Many studies have investigated antibiotic-resistant bacteria in WWTP influent using traditional culture techniques (26) or molecular methods to study ARGs (27). In this study, we quantified *bla*_CTX-M_ group 1 and linked these burdens to specific human microbiome organisms to attempt to distinguish direct human inputs from environmental reservoirs created from either initial seeding from humans or present in free-living, environmental bacteria that are not associated with humans.

We found *bla*_CTX-M_ group 1 gene levels were too high in the influent samples to be wholly derived directly from human inputs. We found single copies of *bla*_CTX-M_ group 1 in the *E. coli* genomes we sequenced (*n* = 9), suggesting one copy represented one organism. However, other studies have reported one to five copies of *bla*_CTX-M_ (28, 29). Even considering up to five copies per cell of *bla*_CTX-M_, the levels detected are not consistent with *bla*_CTX-M_ group 1 signals originating solely from human inputs. The load of *bla*_CTX-M_ group 1 genes across the two WWTP service areas averaged 1.22E + 10 copies, compared with the HF183, which is highly prevalent in human feces (30) and averaged just ~30-fold more at 3.35E + 11 per person per day. When considering actual ratios of organisms rather than copy number, the ratio falls only ~6- to 15-fold more HF183 organisms compared to *bla*_CTX-M_ group 1 organisms. This is based on two to five copies in *Bacteroides dorei* (31, 32) and one copy of *bla*_CTX-M_ group 1. Differential cell loss between *Bacteroides* HF183 organisms, which are expected to degrade quickly, and *Enterobacteriaceae* can further contribute to the disproportionally high signal.

### Conveyance system conditions influence *bla*_CTX-M_ group 1 concentrations

The complex relationships among organisms and the differences we observed between two WWTPs with different travel times suggest that *bla*_CTX-M_ group 1 carrying organisms are not simply transported to the WWTP but rather are highly influenced by the conditions in the pipe system. Wastewater conveyance systems are complex environments with high biomass and fluctuating oxygen levels. Our work and others have demonstrated that *Enterobacteriaceae*, *Acinetobacter*, and *Aeromonas* all appear to be resident organisms (33, 34), which support the hypothesis that *Enterobacteriaceae* and *Aeromonas* that carry *bla*_CTX-M_ group 1 could readily survive, accumulate, and potentially propagate. While *Acinetobacter* was not isolated in our limited culture survey, it has been also reported to carry *bla*_CTX-M_ group 1 and could have the potential for a similar dynamic.

In our Boruta analysis, we observed delivery of *bla*_CTX-M_ group 1 followed a similar dynamic of delivery to the JI WWTP as our human, *E. coli*, and *K. pneumoniae* markers, whereas for the SS system, there was no discernible pattern between *bla*_CTX-M_ group 1 and these bacterial markers (Fig. 2). The SS conveyance system has approximately three times longer travel times and at least twice the km of pipes leading to the WWTP compared to JI conveyance system but serves a similar population. The biological oxygen demand (BOD) was also significantly higher in SS vs JI systems. These conditions can affect the degradation of the human markers, as well as provide different environmental conditions and opportunities for growth for organisms seeded into the conveyance system (35, 36).

### Evidence of growth and accumulation of *Enterobacteriaceae* and *bla*_CTX-M_ group 1 organisms

In JI, the abundances of all markers except the human markers significantly increased in warm sampling periods compared to cold. The observation that the human markers remained constant assured us that the increases in other markers were not influenced by flow or large seasonal changes in degradation dynamics. In SS, the only target that followed the same pattern was *K. pneumoniae*, without a concurrent increase in overall *bla*_CTX-M_ group 1. Since we found that only 25% of the *bla*_CTX-M_ group 1 could be attributed to *K. pneumoniae* host organisms, it is possible that the conditions are less favorable to *E. coli* and other *Enterobacteriaceae*. This finding is consistent with higher BOD conditions in SS WWTP, as well as a separate study of independent samples collected at the same time, which were part of a wastewater surveillance program (12). Multiple studies have observed higher concentrations of cefotaxime-resistant organisms in WWTPs in regions with warmer temperatures (37, 38), or seasons (39, 40), suggesting this is a global phenomenon.

### Sequencing of CTX-M amplicons and identification of microbial host organisms

The amplicon sequencing of *bla*_CTX-M_ groups 1–9 and subsequent Comprehensive Antibiotic Resistance Database (CARD) analysis revealed that group 1 is the primary *bla*_CTX-M_ gene, comprising 63% of the ASVs, with an additional 4.5% matching group 9, along with other minor groups. A large percentage of ASVs did not match known groups, which is consistent with other reports where nearly 50% (13) and 23% (41) of *bla*_CTX-M_ gene sequences were considered novel, which might suggest a reservoir of clinically relevant genes (13). It is noteworthy that these were not clearly group 1 and may not have the same association with clinical strains as we observed with the *bla*_CTX-M_ group 1 isolates we genome sequenced.

One major objective of the study was to determine if the high signal of *bla*_CTX-M_ group 1 was derived from organisms commonly implicated in clinical infections, specifically *E. coli* or *K. pneumoniae*. We found 52% of our cultured *bla*_CTX-M_ group 1 isolates from wastewater were *E. coli* or *K. pneumoniae*, similar to other reports (42). Only 1 of the 15 *bla*_CTX-M_ group 1 isolates was *Aeromonas*, which is consistent with other studies that noted rare occurrence in wastewater sludge samples, and no detection in influent (43). As confirmation that we were not missing *bla*_CTX-M_ group 1 carrying organisms on our selective CHROM agar media, we verified the culture abundance was similar to what was determined with ddPCR.

### Resistance and virulence gene profiles of *E. coli* in wastewater resemble clinical isolates

Wastewater *E. coli* commonly carry clinically important antimicrobial resistance determinants, including ESBL *bla*_CTX-M_ variants and multidrug resistance plasmids that are also frequently detected in hospital-associated isolates (44). Additionally, it has been shown that wastewater-derived ESBL *E. coli* frequently harbor virulence factors characteristic of extraintestinal pathogenic *E. coli*, including genes involved in iron acquisition, adhesion, and toxin production, many of which are also prevalent in clinical isolates (45). In parallel, studies have shown co-occurrence of ARGs and virulence genes in wastewater *E. coli* (46) and generally in *Enterobacteriaceae* (47), suggesting a physical linkage that facilitates their co-transfer and co-selection. This genetic coupling may imply that the selective pressures in the wastewater environment may not only maintain or enrich the resistance but also indirectly select for strains with enhanced virulence potential. In addition, recent whole-genome sequencing studies have shown that *K. pneumoniae* in wastewater, also found to be a major contributor to the *bla*_CTX-M_ group 1 in our study, overlap in both antibiotic resistance gene and virulence gene content with clinical isolates (48).

In this study, we link high *bla*_CTX-M_ group 1 signals with the host organisms and their virulence potential to demonstrate that *E. coli*, and likely *K. pneumoniae*, carrying *bla*_CTX-M_ group 1 constitutes a major reservoir of potential pathogens which can contribute to a transmission cycle (49, 50). Approximately 3% of *E. coli* isolated from raw influent samples contained *bla*_CTX-M_ group 1, slightly lower than the ratio of the *E. coli* and *bla*_CTX-M_ group 1 determined by PCR-based methods in recent samples, and in agreement with past studies where a prevalence of 2.0 to 0.35% for ESBL *E. coli* was reported (51, 52). Collectively, these findings highlight wastewater as an important ecological interface where clinically relevant *E. coli* populations and their associated virulence and resistance gene repertoires persist and potentially disseminate.

### Applications and limitations of WWTP surveillance data for AMR

Unlike monitoring for human-specific viruses, which do not replicate outside the host, using wastewater to monitor for antibiotic-resistant bacteria is complicated by the ability to grow as free-living organisms outside the host, the potential for horizontal gene transfer among other environmental organisms, and the challenge of linking resistance genes to a pathogenic organism (49, 53). A discordance between what is observed in the clinical setting versus what can be recovered in wastewater has been noted in other studies (54). Monitoring of wastewater may be most useful for emerging ARG targets that have not experienced widespread dissemination into wastewater systems, such as New Delhi metallo-beta-lactamase (NDM) (12). In addition, long-term accumulation of targets of interest in the human population might be reflected in increasing and eventually ubiquitous occurrence in wastewater, and observing these dynamics could provide insights into the rate of accumulation and magnitude of environmental reservoirs. Challenges in long-term monitoring include the necessity to have comparable results over a span of years or reliable methods to archive samples to be able to normalize to degradation or evaporation, such as the addition of internal standards. The ability to reliably quantify these targets over time should be considered in the overall frameworks proposed for antimicrobial resistance monitoring (49, 50, 53).

### Conclusion

Our study adds to the growing body of evidence that wastewater conveyance systems, which comprise tens of thousands of km of pipes in a major city, can serve to propagate ESBL bacteria and provides insight into the successive accumulation of increasingly concerning antibiotic-resistant organisms. The increasing occurrence and distribution of CTX-M group 1 over the past 25 years, as well as evidence of the large reservoirs seen in community wastewater, is an indication of what might be expected with carbapenem resistance genes in *E. coli* and other *Enterobacteriaceae*. While wastewater surveillance has been proposed as a means to understand the burden in the human population, our data would suggest that for antibiotics that have been used in the human population for some time, the burden in community wastewater could be higher than reservoirs in humans. Our observation of virulence gene profiles supports inputs into sewers of clonal strains and propagation rather than gene transfer *in situ* spreading CTX-M genes to *E. coli* that are not necessarily primed to be human pathogens. The One Health approach to addressing the antibiotic crisis needs to consider wastewater systems in urban areas as a major reservoir of virulent, resistant organisms (49, 50).

## MATERIALS AND METHODS

### Selection of assays for antibiotic resistance genes and bacterial markers

We used previously reported assays (55) for *bla*_CTX-M_ group 1 genes, which capture the most clinically relevant CTX-M genes, including CTX-M-15 (Table 5).

**TABLE 5:** Primers and probes used in this study

Primer name	Sequence 5′ – 3′	Target	Method	Reference
F CTX-M con	5′ATGTGCAGYACCAGTAARGTKATGGC3′	CTX-M groups 1–9	ddPCR	(55)
R CTX-M con	5′ATCACKCGGRTCGCCAGGRAT3′	CTX-M groups 1–9	FAM-BHQ1	
CTX-M-1 P	5′CCCGACAGCTGGGAGACGAAACGT3′	CTX-M group 1		
HF183F	5′ATC ATG AGT TCA CAT GTC CG3′	Human *Bacteroides*	qPCR	(56)
BacHum241R	5′CGT TAC CCC GCC TAC TAT CTA ATG3′	16s rDNA	MGBNFQ	
BacHum193p	5′TCC GGT AGA CGA TGG GGA TGC GTT3′			
899Lachno3F	5′CAA CGC GAA GAA CCT TAC CAA A3′	*Closdriales*	qPCR	(57)
932Lachno3R	5′CCC AGA GTG CCC ACC TTA AAT3′	*Lachnospiraceae*	MGBNFQ	
1065Lachno3p	FAM 5′CTC TGA CCG GTC TTT AAT CGG A3′	16s rDNA		
uidA1663F	5′GCG ACC TCG CAA GGC ATA3′	*E. coli*	qPCR	(58)
uidA1790R	5′GAT TCA TTG TTT GCC TCC CTG CTG CG3′	β-glucuronidase	MGBNFQ	
uidA1729p	FAM 5′TGC AGC AGA AAA GCC GCC GAC TTC GG3′			
kheF	5′CGATGCTACTTATCCCGACA3′	*K. pneumoniae*	qPCR	(59)
kheR	5′AGCCGGTTGAGACGTAAAC3′	Hemolysin gene	FAM-BHQ1	
khep	5′CCGATTGAAAAACGCTCCGGGC3′			

### Sewage sample collection, filtration, and nucleic acid extraction

We quantified concentrations of *bla*_CTX-M_ group 1, human fecal markers (HF183 and Lachno3), *E. coli*, and *K. pneumoniae* in 201 raw influent samples from 4 WWTPs (Table 1), which were part of previous projects (21, 60), including the Wisconsin SARS-CoV-2 wastewater surveillance program (August 2020–July 2024) (11). Samples were 24-h flow-weighted composites of influent from the four WWTPs, collected by the Milwaukee Metropolitan Sewerage District (MMSD) and the municipalities of Green Bay and Racine. Sample processing occurred within 24 h of collection. A volume of 25 mL was filtered onto a 0.22 µm mixed cellulose ester filter (47 mm diameter; Millipore, Billerica, MA, USA), and the filters were stored at −80°C until DNA extraction. Immediately before extraction, the frozen filters were shattered into small pieces with a metal spatula. DNA was extracted using the MPBIO FastDNA SPIN Kit for Soil (MP Biomedicals, Santa Ana, CA, USA) according to manufacturer instructions, except for adding a 0.2 µg/µL salmon sperm solution to serve as a DNA recovery marker. DNA was eluted in 150 µL of nuclease-free water and stored at −20°C until PCR experiments.

### Digital droplet PCR for *bla*_CTX-M_ group 1

Digital droplet PCR was done with the ddPCR Supermix for Probes (No dUTP) (Bio-Rad, Hercules, CA, USA) and the droplet data were processed using the QuantaSoft Software, version 1.7, for the Bio-Rad QX200 Droplet Digital System (Bio-Rad, Hercules, CA, USA). Some samples were diluted 1:50 if their initial run was unsuccessful due to high DNA concentrations.

### Quantitative PCR for bacterial markers

Quantitative PCR (qPCR) was done using the Applied Biosystems StepOne Plus Real-Time PCR System Thermal Cycling Block (Applied Biosystems; Foster City, CA, USA) with TaqMan hydrolysis probe chemistry. All DNA samples were diluted 1:50. Primers and probes from previous publications were used for the human *Bacteroides* (HB) assay (61, 62), which targets the human-associated HF183 bacterial cluster (56). The HB assay reported in Templar et al*.* (56) uses primers from a prior paper (63), except that the HF183F was used as the forward primer (64). Lachno3 assay and protocol were sourced from previous studies (57). Assays for the presumptive human hosts, *K. pneumoniae* and *E. coli*, were also sourced from prior publications (58, 59).

To assess DNA recovery and viability, we performed Sketa qPCR and Qubit DNA quantification analysis on a large subset of samples. Samples undergoing Sketa tests had salmon sperm added during the extraction process to ascertain extraction efficiency. Samples with abnormally low marker abundances with low DNA concentrations were removed from the data set due to low recovery.

### Diversity of *bla*_CTX-M_ genes in wastewater samples using amplicon sequencing

The *bla*_CTX-M_ group 1 amplicon sequencing was performed on 14 wastewater samples from four Wisconsin WWTPs in three cities, Milwaukee (JI and SS), Racine (RAC), and Green Bay (PLT). DNA was extracted and amplified using the *bla*_CTX-M_ group 1–9 primers (Table 5). PCR products were sequenced on an Illumina Miseq using 2 × 300 bp paired-end sequencing at the Great Lakes Genomics Center. We used BLAST (65) to identify *bla*_CTX-M_ groups, and amplicons that were not CTX-M were removed from analysis.

### Culture of ESBL isolates from community wastewater samples and identification of *bla*_CTX-M_ genes

We plated dilutions of four untreated wastewater influent samples, collected on 1 July and 7 July 2025 from JI and SS, respectively, on ESBL CHROMagar plates for selective growth of ESBL producers. Plates were incubated for 18 h at 36°C, and total colonies were counted based on phenotypic characteristics as presumptive *E. coli* (EC, pink to purple), presumptive *Klebsiella*/*Enterobacter*/*Citrobacter* (KEC, blue), and presumptive *Acinetobacter* (AC, white). A total of 48 colonies were picked and screened with primers and probes listed in Table 5 (specifically *bla*_CTX-M_ group 1, *E. coli*, and *K. pneumoniae*). The *bla*_CTX-M_ amplicons from isolates were generated using the *bla*_CTX-M_ group 1–9 forward and reverse primers (Table 5), and full-length 16S rRNA genes were generated using 8F and 1492R primers. Amplicons were sent to the Roy J. Carver Biotechnology Center, University of Illinois at Urbana-Champaign, for Sanger sequencing.

Full-length 16S rRNA gene sequences of isolates were classified for taxonomy using MAPseq (v2.1.1) (66). BLAST analysis was performed to identify those for which genus level was not achievable. In all, 45/48 of the isolates were sequenced successfully for 16S rRNA gene, and 23/48 had *bla*_CTX-M_ amplicons produced. Unique *bla*_CTX-M_ sequence types were analyzed in the Comprehensive Antibiotic Resistance Database (CARD) to compare with known *bla*_CTX-M_ subtypes. The output of CTX-M enzymes with 100% identity was analyzed using the Beta-Lactamase Database for subfamily classification.

To compare the representation of our cultured organisms with our ddPCR results, we counted all plates for each isolate type, multiplied by the *bla*_CTX-M_ group 1% positives confirmed by PCR screening and sequencing, and back-calculated to units of Log_10_CN/100mL for comparison to abundances observed in ddPCR. Our estimated *bla*_CTX-M_ group 1 abundance at JI and SS was 6.09 and 5.93 Log_10_CN/100mL, respectively.

### Screening of *E. coli* from community wastewater on non-selective media for prevalence of *bla*_CTX-M_ group 1

To determine the prevalence of *bla*_CTX-M_ group 1 in *E. coli* as a host organism, we plated dilutions of wastewater samples from JI and SS on MOD-mTEC plates to achieve distinct colonies that could be screened by colony PCR. The plates were incubated at 35°C for 2 h and 44.5°C for 21 h before colonies were counted. Colonies (JI = 56; SS = 96) were suspended in 50 µL of nuclease-free water, boiled at 100°C for 15 min, and diluted 1:10 for qPCR. For screening, we defined samples with Ct < 35 as positive for *bla*_CTX-M_ group 1. The *E. coli* qPCR assay was used as a positive control to confirm species identity and sufficient cell abundance in the pellet for detection.

### Isolation and whole-genome sequencing of *E. coli* from wastewater

We selectively isolated *E. coli* using MOD-mTEC agar supplied with 4 mg/mL cefotaxime for whole-genome sequencing on an Illumina Miseq. Whole-genome sequences of *E. coli* isolates were generated by this study (*n* = 24) and obtained from published data (*n* = 72) for clinical genomes, NCBI BioProject accession PRJNA288601; *n* = 24 for wastewater genomes isolated without selection, genome assembly from NCBI BioProject accessions PRJNA514245 and PRJNA790841) (67–69). Collected genomes were assigned to biological groups of clinical and wastewater sources; clinical samples were further grouped by their available information of isolation source, including abdomen/bile (*n* = 9), blood (*n* = 6), rectal/perianal (*n* = 13), sputum/BAL (*n* = 3), urine (*n* = 30), and other/unknown sources (*n* = 11). Genome sequences generated by this study were adapter and quality trimmed using TrimGalore (v0.6.7, https://github.com/FelixKrueger/TrimGalore/) and were assembled using megahit (v1.2.9) (70). Assembled contigs were processed with Anvi’o (v8) (71) to annotate beta-lactam resistant genes and screened using VirulenceFinder (72) to identify virulence genes. The beta-lactam resistance genes were normalized as (raw gene counts / sample number in each group × 100), and the pattern of virulence factors was evaluated using principal component analysis (PCA). Virulence genes were also compared between selectively isolated wastewater *bla*_CTX-M_+ isolates and non-selective isolates (all *bla*_CTX-M_−) to identify genes enriched in these two groups. *E. coli* sequence types (STs) were identified using the *mlst* tool (https://github.com/tseemann/mlst) with the ecoli_achtman_4 scheme.

### Statistical analyses and rigor

The abundances of each target were log-transformed before analysis. Analyses were performed using R version 4.4.1. After performing the Shapiro-Wilk normality test on each subset of samples, all except one showed non-normally distributed data. We then used Spearman correlations to examine the relationships among *bla*_CTX-M_ group 1 and human and presumptive host markers. Due to small sample sizes (*n* < 30), we also tested Kendall Tau correlations instead of Spearman, but the significance of the relationships remained constant, and their *r* values were not different. Linear regression was used to determine if *bla*_CTX-M_ group 1, *E. coli*, or *K. pneumoniae* were increasing over time.

Boruta analysis (73) was implemented to understand which factors most influenced *bla*_CTX-M_ group 1 abundances, both across and within each WWTP. Factors under consideration included WWTP, flow (million gallons per day [MGD]), and the human/presumptive host bacterial markers.

## Data Availability

The amplicon and whole-genome sequencing data generated in this study have been deposited in the NCBI database under BioProject accession no. PRJNA1457535.

## SUPPLEMENTAL MATERIAL

Data Set S1 aem.00890-26-s0001.xlsx Sequence types and ARG contents of genomes used in this study.

Supplemental material aem.00890-26-s0002.pdf Tables S1 to S6; Fig. S1 to S3.

ASM does not own the copyrights to Supplemental Material that may be linked to, or accessed through, an article. The authors have granted ASM a non-exclusive, world-wide license to publish the Supplemental Material files. Please contact the corresponding author directly for reuse.
