# Supplementary material for: Dynamics and virulence of Enterobacteriaceae reservoirs harboring blaCTX-M group 1 in community wastewater
Source: Appl Environ Microbiol. 2026 Sep 8;92(10):e00890-26. doi: 10.1128/aem.00890-26 (PMC13689389; doi:10.1128/aem.00890-26)
Supplement: Supplementary file 1 [file aem.00890-26-s0002.pdf]

Dynamics and virulence of Enterobacteriaceae reservoirs harboring *bla*$_{CTX-M}$ group 1 in community wastewater systems

Kieyarrah J. Dennis[1], Shuchen Feng[1], Melissa K. Schussman[1], Melinda J. Bootsma, and Sandra L. McLellan[1]*

[1] School of Freshwater Sciences, University of Wisconsin- Milwaukee

600 E Greenfield Ave, Milwaukee, WI 53204

* Corresponding author: mclellan@uwm.edu

Supplemental Information

Table S1. Mean, standard deviation (SD), and relative standard deviation (%SD) of all markers at four WWTPs

Table S2. T-test values of marker increase from cold to warm months

Table S3. Organisms occurring as less than 5% of NCBI matches to each ASV

Table S4. Overview of the 48 colonies isolated on ESBL CHROMagar™ from raw influent samples.

Table S5. Distribution of *bla*$_{CTX\text{-}M}$ subtypes in bla$_{CTX-M}$ + clinical and wastewater genomes.

Table S6. Sample list for four WWTPs

Figure S1. Linear regression of blaCTX-M group 1 (A), E. coli (B), and K. pneumoniae (C) over a 10-year period at JI (Jones Island) and SS (South Shore) WWTPs.

Figure S2. Spearman's correlation matrices of log_{10}-transformed (A) JI (n = 94), (B) SS (n = 67), and (C) JI daily (n = 24) marker values.

Figure S3. Heat map of virulence gene profiles in clinical and wastewater *E. coli* genomes.

Table S1. Mean, standard deviation (SD), and relative standard deviation (%SD) of all markers at four WWTPs.

City	Target	Mean (Log_{10}CN/100mL)	SD (Log_{10}CN/100mL)	% SD
Milwaukee JI	CTX-M group 1	6.00	0.58	9.66
Milwaukee JI	HF183	7.72	0.33	4.29
Milwaukee JI	Lachno3	7.64	0.49	6.41
Milwaukee JI	*E. coli*	6.37	0.31	4.93
Milwaukee JI	*K. pneumoniae*	6.68	0.48	7.21
Milwaukee SS	CTX-M group 1	6.60	0.70	10.60
Milwaukee SS	HF183	7.87	0.66	8.37
Milwaukee SS	Lachno3	7.81	0.80	10.30
Milwaukee SS	*E. coli*	6.64	0.52	7.90
Milwaukee SS	*K. pneumoniae*	7.18	0.62	8.62
Racine RAC	CTX-M group 1	5.94	0.42	7.08
Racine RAC	HF183	7.74	0.29	3.78
Racine RAC	Lachno3	7.73	0.39	4.99
Racine RAC	*E. coli*	6.39	0.32	5.05
Racine RAC	*K. pneumoniae*	6.34	0.40	6.33
Green Bay PLT	CTX-M group 1	6.57	0.29	4.39
Green Bay PLT	HF183	7.74	0.14	1.82
Green Bay PLT	Lachno3	7.72	0.23	2.97
Green Bay PLT	*E. coli*	6.74	0.25	3.64
Green Bay PLT	*K. pneumoniae*	6.62	0.36	5.49

Table S2. T-test values of marker increase from cold to warm months

Marker	WWTP	
	JI	SS
bla$_{CTX-M}$ group 1	t(61) = -2.81, p = 0.0066	t(53) = 0.69, p = 0.49
HF183	t(49) = -1.05, p = 0.30	t(52) = 0.03, p = 0.98
Lachno3	t(49) = -1.36, p = 0.18	t(45) = -1.54, p = 0.13
E. coli	t(49) = -6.24, p = 9.97E-08	t(36) = -3.34, p = 0.0019
K. pneumoniae	t(57) = -6.47, p = 2.43E-08	t(38) = -3.23, p = 0.0025

Table S3. Organisms occurring as less than 5% of NCBI matches to each ASV

ASV_1	ASV_2	ASV_8	ASV_15	ASV_17	ASV_21	ASV_23
A. baumannii<br>*A. caviae*<br>*bacterium*<br>*C. braakii*<br>*C. freundii*<br>*C. koseri*<br>*C. portucalensis*<br>*Citrobacter sp. EC23*<br>*E. ictaluri*<br>*E. asburiae*<br>*E. chuandaensis*<br>*E. hormaechei*<br>*H. parainfluenzae*<br>*K. aerogenes*<br>*K. michiganensis*<br>*K. oxytoca*<br>*K. variicola*<br>*K. cryocrescens*<br>*M. morganii*<br>*P. mirabilis*<br>*P. huaxiensis*<br>*P. rettgeri*<br>*Providencia sp. PROV001*<br>*P. stuartii*<br>*P. aeruginosa*<br>*R. ornithinolytica*<br>*R. planticola*<br>*S. enterica*<br>*S. dysenteriae*<br>*S. flexneri*<br>*S. sonnei*<br>*S. alvi*	*P. agglomerans*	*C. youngae*<br>*K. oxytoca*	*A. baumannii*<br>*C. freundii*<br>*C. koseri*<br>*E. cloacae*<br>*E. hormaechei*<br>*E. fergusonii*<br>*Escherichia sp.*<br>*K. aerogenes*<br>*K. oxytoca*<br>*K. ascorbata*<br>*M. morganii subsp. morganii*<br>*Phytobacter sp. MRY16-398*<br>*P. rettgeri*<br>*P. aeruginosa*<br>*R. ornithinolytica*<br>*S. enterica*<br>*S. flexneri*<br>*uncultured bacterium*<br>*V. cholerae*	*K. pneumoniae*	*A. caviae*<br>*A. hydrophila*<br>*C. gilardii*<br>*P. alloputida*<br>*P. chengduensis*<br>*P. fluorescens*<br>*P. juntendi*<br>*P. plecoglossicida*<br>*Pseudomonas sp. PS1(2021)*<br>*S. maltophilia*<br>*uncultured bacterium*	*C. braakii*<br>*C. cronae*<br>*C. freundii*<br>*C. koseri*<br>*E. cloacae*<br>*E. hormaechei*<br>*E. roggenkampii*<br>*E. albertii*<br>*K. aerogenes*<br>*L. adecarboxylata*<br>*M. morganii*<br>*Salmonella sp.*<br>*S. dysenteriae*<br>*S. flexneri*<br>*S. sonnei*<br>*uncultured bacterium*<br>*V. cholerae*<br>*V. parahaemolyticus*<br>*V. vulnificus*

Table S4. Overview of the 48 colonies isolated on ESBL CHROMagar™ from raw influent samples.

Isolate	Genus (species when possible)	ASV*	KHE_qPCR	EC_qPCR	CARD CTX-M Grouping Results*
EC-14	Escherichia	ASV_1	no	yes	1
EC-15	Escherichia	ASV_1	no	yes	1
EC-17	Escherichia	ASV_1	no	yes	1
EC-8	Escherichia	ASV_1	no	yes	1
KEC-10	Aeromonas	ASV_1	no	no	1
KEC-11	K. pneumoniae	ASV_1	yes	no	1
KEC-13	Raoultella	ASV_1	no	no	1
KEC-14	Klebsiella/Raoultella	ASV_1	no	no	1
KEC-16	Enterobacter	ASV_1	no	no	1
KEC-18	Klebsiella	ASV_1	yes	no	1
KEC-19	Klebsiella	ASV_1	yes	no	1
KEC-2	Klebsiella	ASV_1	no	no	1
KEC-4	Klebsiella	ASV_1	yes	no	1
KEC-8	Enterobacter	ASV_1	no	no	1
KEC-9	Raoultella	ASV_1	no	no	1
EC-12	Escherichia	ASV_30	no	yes	9, 1
EC-16	Escherichia	ASV_30	no	yes	9, 1
EC-19	Escherichia	ASV_30	no	yes	9, 1
EC-2	Escherichia	ASV_30	no	yes	9, 1
EC-3	Escherichia	ASV_30	no	yes	9, 1
EC-13	Escherichia	ASV_38	no	yes	9
KEC-1	Klebsiella/Raoultella/Kluyvera	ASV_52	no	no	9
KEC-12	Kluyvera/Klebsiella	n/a	no	no	n/a
EC-1	n/a	n/a	no	no	n/a
EC-7	Aeromonas	n/a	no	no	n/a
EC-18	Escherichia	n/a	no	yes	n/a
EC-20	Aeromonas	n/a	no	no	n/a
EC-11	Aeromonas	n/a	no	no	n/a
KEC-3	Klebsiella/Raoultella	n/a	no	no	n/a
EC-5	Aeromonas	n/a	no	no	n/a
EC-6	Citrobacter	n/a	no	no	n/a
EC-9	Citrobacter	n/a	no	no	n/a
EC-10	Aeromonas	n/a	no	no	n/a
KEC-5	Enterobacter	n/a	no	no	n/a
KEC-7	Klebsiella	n/a	yes	no	n/a
KEC-15	Aeromonas	n/a	no	no	n/a
KEC-17	Aeromonas	n/a	no	no	n/a

KEC-20	Raoultella	n/a	no	no	n/a
A-1	n/a (Acinetobacter)	n/a	no	no	n/a
A-2	Acinetobacter baumannii	n/a	no	no	n/a
A-4	Pseudomonas	n/a	no	no	n/a
A-5	Acinetobacter baumannii	n/a	no	no	n/a
A-6	Pseudomonas	n/a	no	no	n/a
A-7	Acinetobacter	n/a	no	no	n/a
A-8	Acinetobacter	n/a	no	no	n/a
KEC-6	Enterobacter	n/a	no	no	n/a
A-3	Pseudomonas	n/a	no	no	n/a
EC-4	Citrobacter	n/a	no	no	n/a

* n/a, not applicable.

Table S5. Distribution of *bla_{CTX-M}* subtypes in *bla_{CTX-M}*+ clinical and wastewater genomes.

**bla_{CTX-M} group**	**bla_{CTX-M} subtype**	**# in clinical isolates**	**# in wastewater isolates**
1	CTX-M-15	112	5
	CTX-M-3	2	0
	CTX-M-55	14	4
9	CTX-M-14	10	0
	CTX-M-27	2	0
	CTX-M-65	2	0
Not described	99% identical to CTX-M-15	2	0

Table S6. Sampling schematic for four WWTPs (N =268).

	JI	SS	PLT	RAC
**2013**	9	3		
**2014**	11	2		
**2015**	11	10		
**2016**	12	7		
**2017**	13	10		
**2018**	10	7		
**2019**	8	6		
**2020**	12	12		
**2021**	10	10		
**2023**	20	20	20	20
**2024**	25 (daily)			
**Total**	**141**	**87**	**20**	**20**

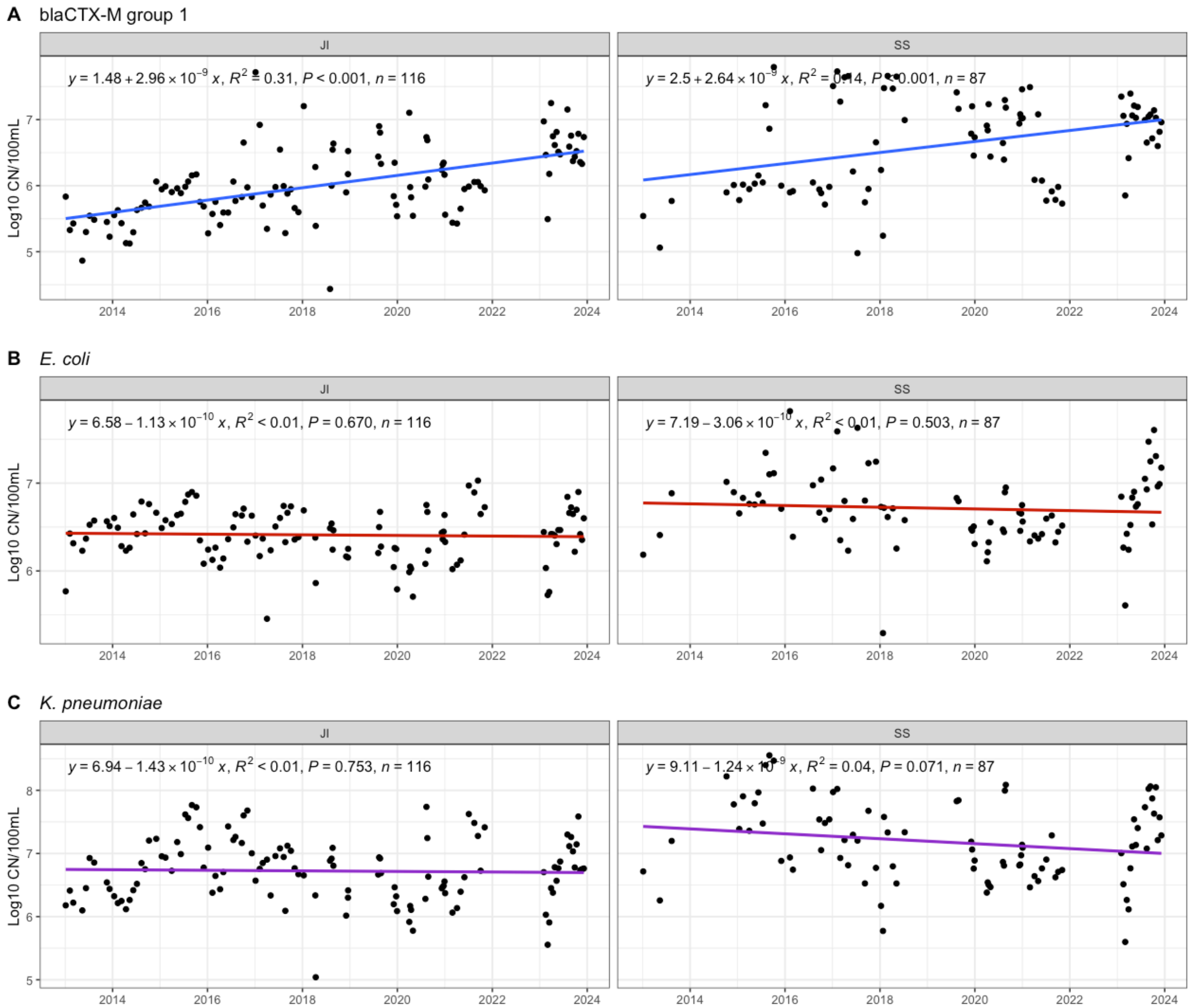

Figure S1. Linear regression of blaCTX-M group 1 (A), E. coli (B), and K. pneumoniae (C) over a 10-year period at (Jones Island (JI) and South Shore (SS) WWTPs.

A JI WWTP over 10 years

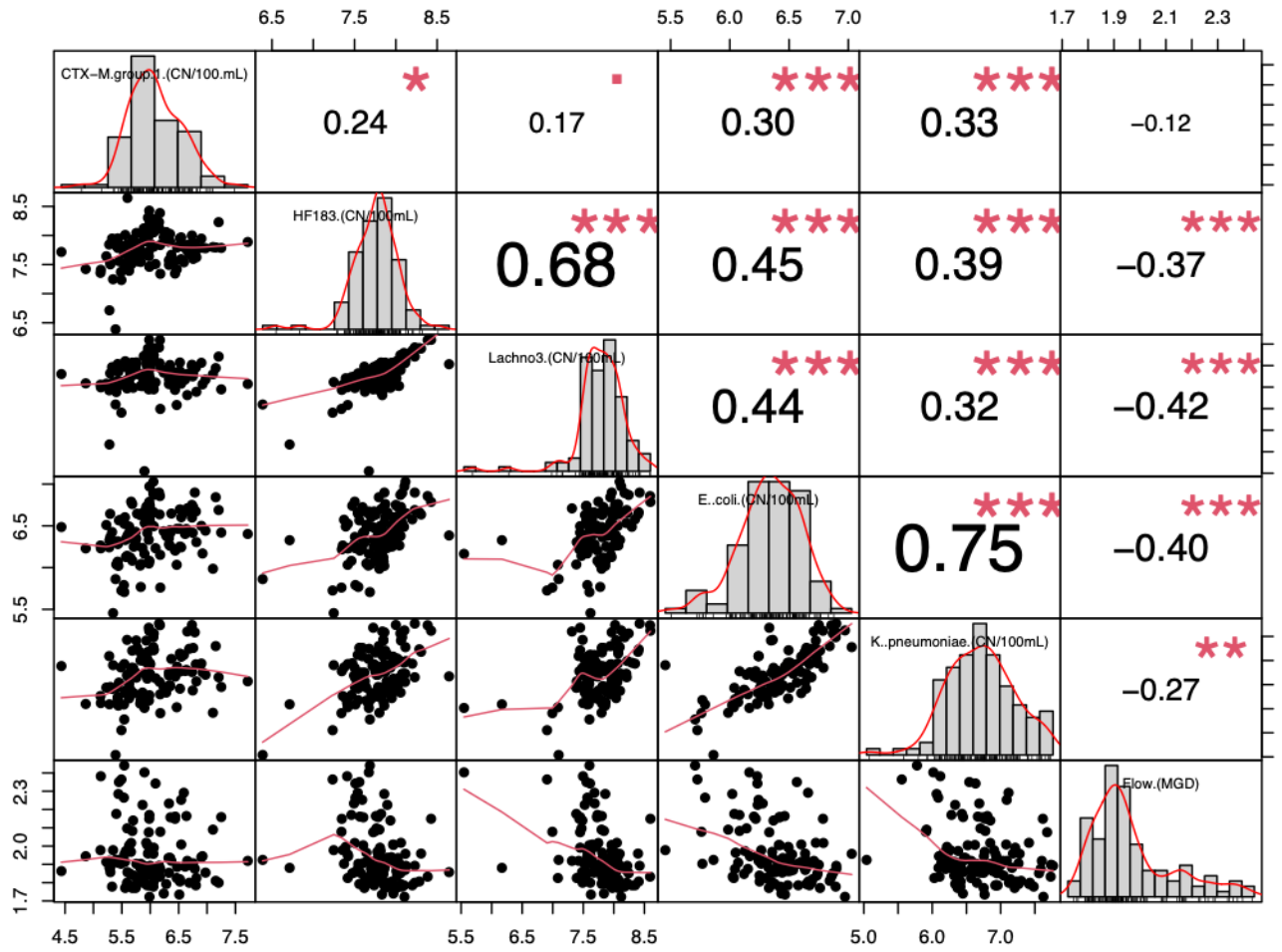

B SS WWTP over 10 years

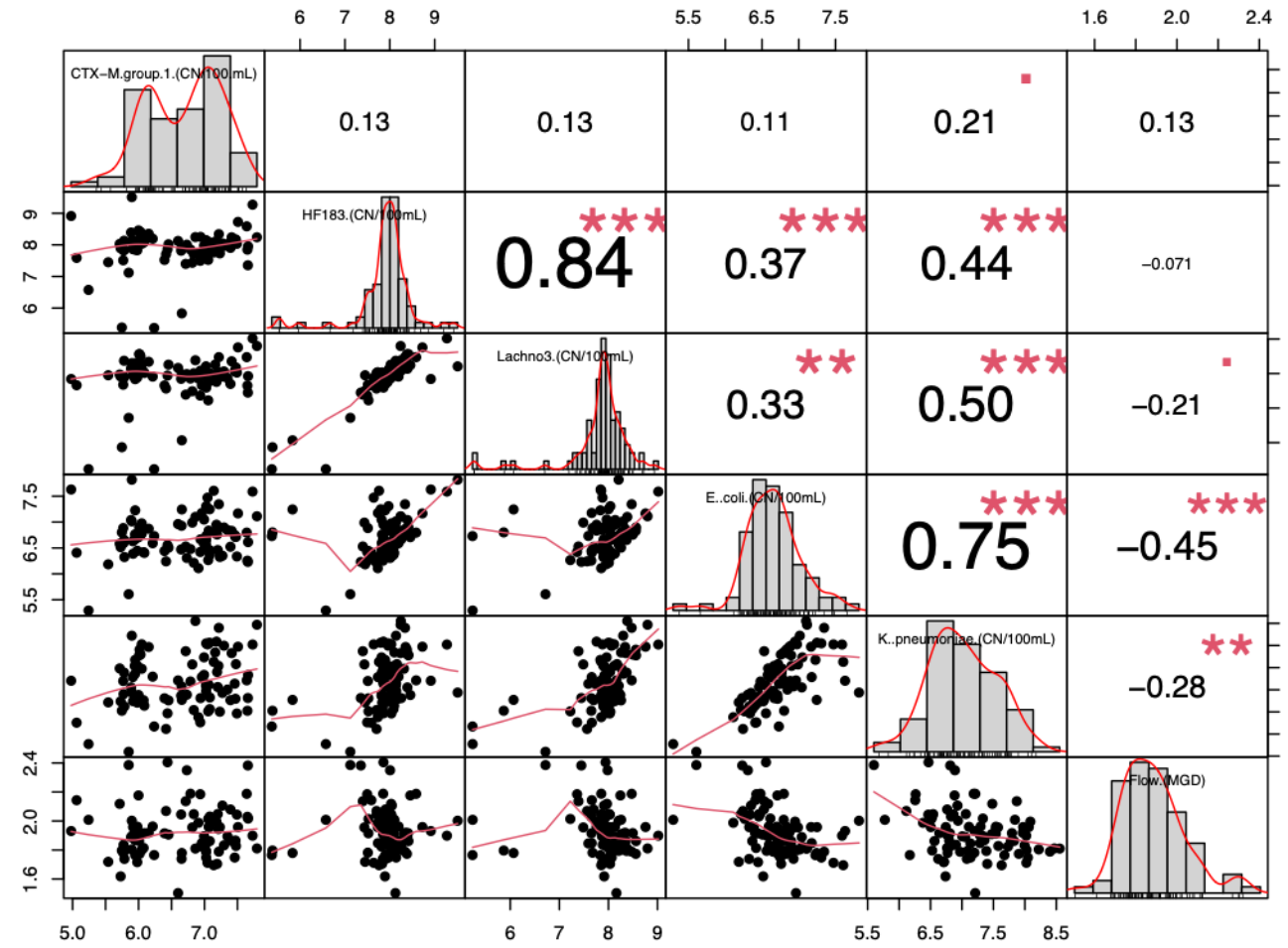

C JI WWTP daily samples

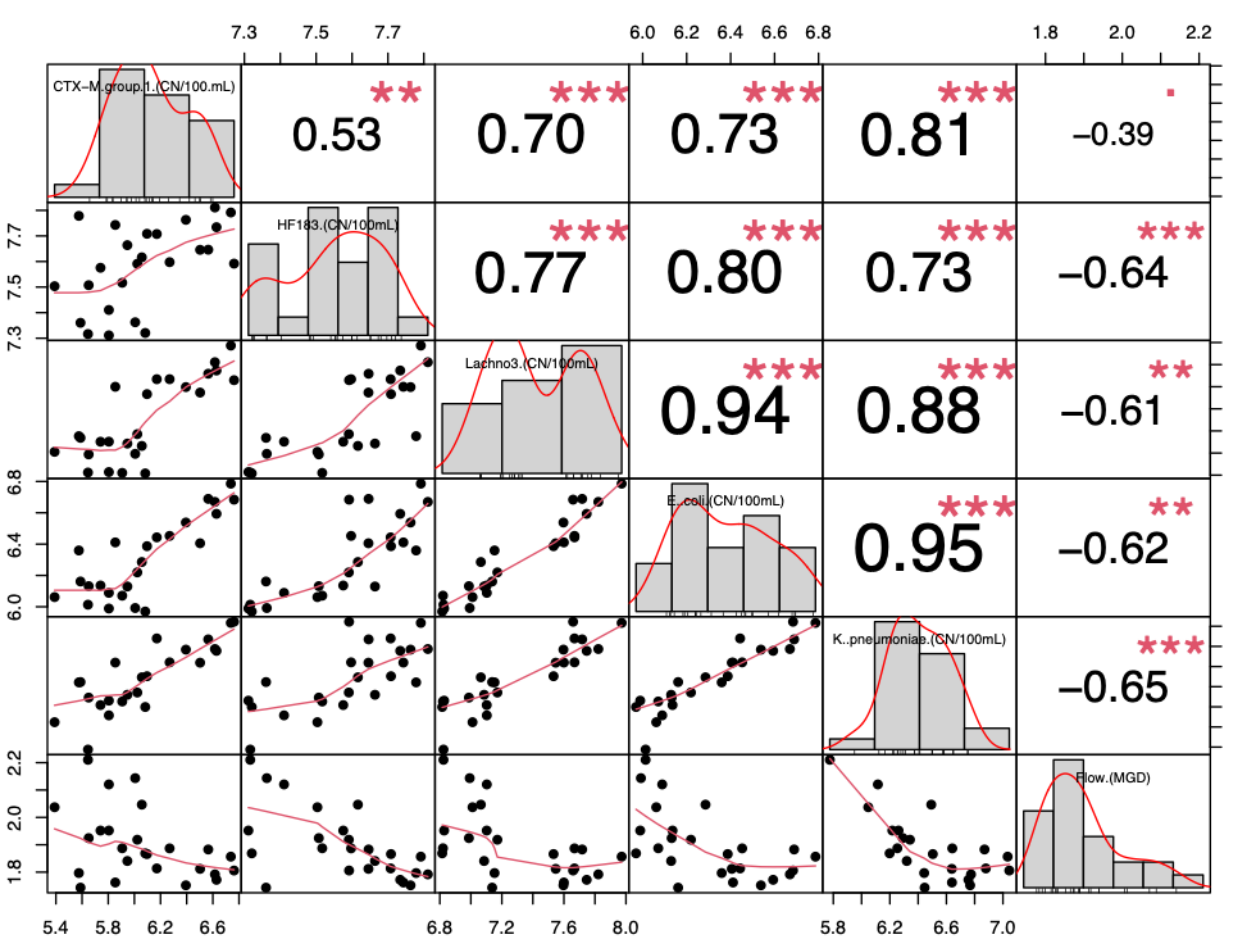

Figure S2. Spearman's correlation matrices of log_{10}-transformed (A) JI (n = 94), (B) SS (n = 67), and (C) JI daily (n = 24) marker values. Marker or flow are indicated on center diagonal. Right upper diagonal shows spearmans rho values and left lower diagonal show plots of the data.

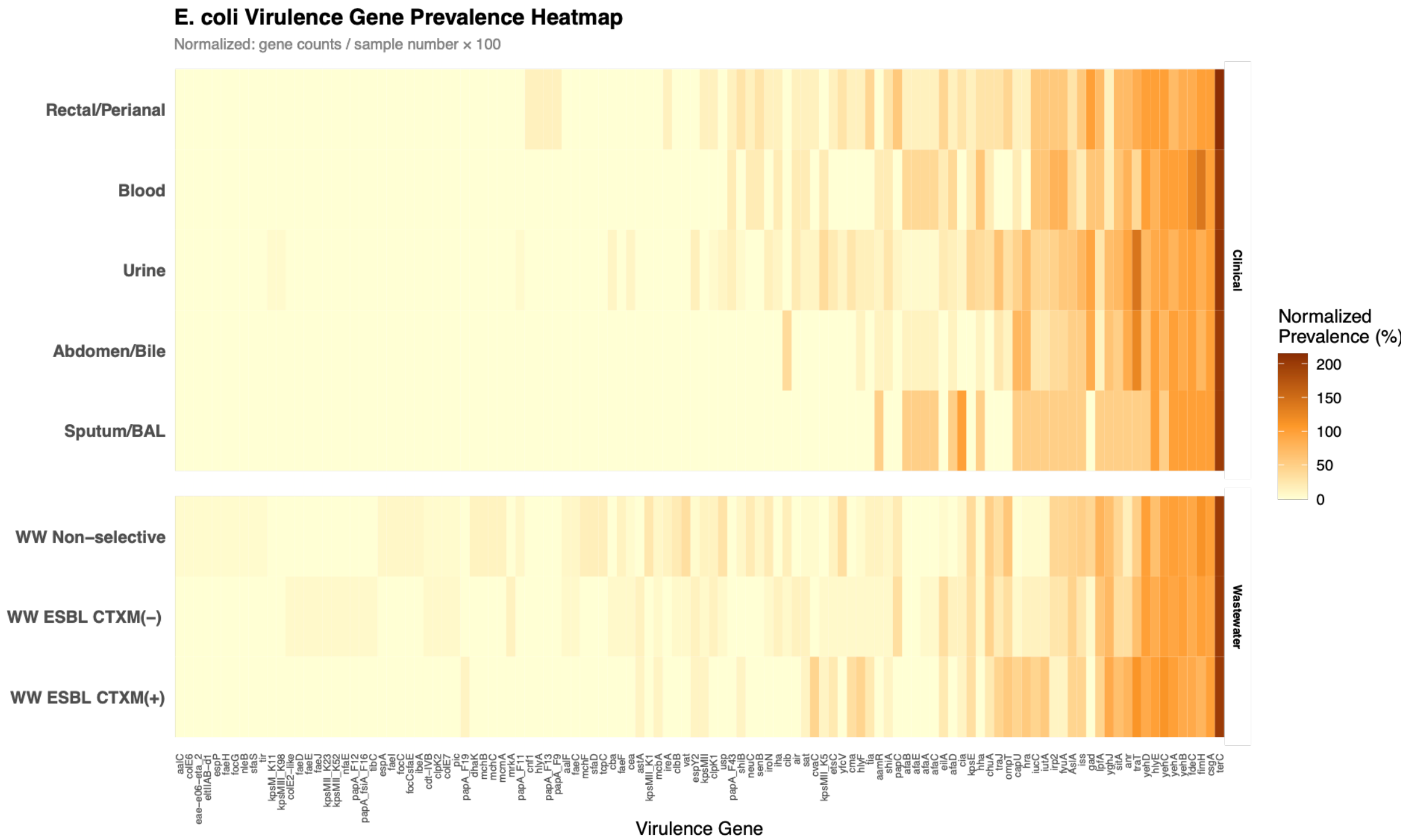

Figure S3. Heat map of virulence gene profiles in clinical and wastewater *E. coli* genomes.
